# Insights into potential multi-target protective effects of Tanshinone IIA in steroid-induced osteonecrosis of the femoral head: evidence from integrated metabolomics and proteomics

**DOI:** 10.3389/fendo.2026.1991120

**Published:** 2026-10-19

**Authors:** Wei Li, Ning Xu, Jin-Lian Chai, Bo-Chun Zhang, Yan-Chen Liang, Gang Li, Xue-Zhen Liang

**Affiliations:** 1 First College of Clinical Medicine, Shandong University of Traditional Chinese Medicine, Jinan, Shandong, China; 2 Department of General Medicine, The 960th Hospital of the PLA Joint Logistic Support Force, Jinan, Shandong, China; 3 College of Traditional Chinese Medicine, Shandong University of Traditional Chinese Medicine, Jinan, Shandong, China; 4 Orthopaedic Microsurgery, Affiliated Hospital of Shandong University of Traditional Chinese Medicine, Jinan, Shandong, China; 5 The Second Affiliated Hospital of Shandong University of Traditional Chinese Medicine, Jinan, Shandong, China

**Keywords:** key protein, metabolomics, proteomics, steroid-induced osteonecrosis of the femoral head, Tanshinone IIA

## Abstract

**Objective:**

To systematically evaluate the interventional effects of Tanshinone IIA on a rat model of steroid-induced osteonecrosis of the femoral head (SONFH) based on metabolomics and proteomics technologies, and to elucidate its multi-target regulatory mechanisms.

**Methods:**

Twenty-four SD rats were randomly divided into a Control group, a model group, and a Tanshinone IIA group, with eight rats in each group. Micro-CT, HE staining, and TRAP staining were used to assess the bone microarchitecture and pathological changes of the femoral head. UPLC-MS/MS-based untargeted metabolomics combined with DIA quantitative proteomics were employed to analyze the metabolite and protein expression profiles of fecal and femoral head samples. Differential metabolites and differentially expressed proteins were screened, followed by KEGG pathway enrichment analysis and Pearson correlation analysis.

**Results:**

Micro-CT showed that the bone volume fraction in the Tanshinone IIA group rebounded significantly compared with the model group, and the trabecular arrangement tended to be orderly. HE staining revealed alleviated adipocyte infiltration, and TRAP staining demonstrated a reduction in osteoclast number. Metabolomics revealed that xanthine, hypoxanthine, 2-oxopentanedioic acid, and succinic semialdehyde were markedly elevated in the model group, whereas these metabolites returned toward normal levels after Tanshinone IIA intervention. KEGG enrichment mainly involved purine metabolism, 2-oxocarboxylic acid metabolism, and amino acid metabolic pathways. Proteomics indicated that differentially expressed proteins in the model group were enriched in the Hippo, TGF-β, and Wnt signaling pathways, whereas Tanshinone IIA intervention primarily affected aminoacyl-tRNA biosynthesis and lipid metabolism pathways. Pearson correlation analysis identified Bmp6, Smoc2, Ihh, and Enpp2 as core candidate proteins.

**Conclusion:**

These findings suggest that Tanshinone IIA may be associated with improved bone microarchitecture and reduced fatty infiltration in the SONFH rat model. The observed changes were associated with alterations in purine and energy metabolism, the Hippo/TGF-β/Wnt signaling pathways, and candidate proteins such as Bmp6 and Ihh. These results indicate potential multi-target associations at three levels—bone protection, metabolic alterations, and signaling network associations—providing preliminary correlative multi-omics evidence for further investigation in SONFH.

## 1 Introduction

Steroid-induced osteonecrosis of the femoral head (SONFH) is a severe bone complication that occurs after long-term or high-dose glucocorticoid administration and is a major cause of disability in young patients (1). With the widespread use of glucocorticoids in autoimmune diseases, organ transplantation, and tumor chemotherapy, the incidence of SONFH has been increasing annually, and the age of onset is tending to be younger, imposing a heavy burden on patients’ families and healthcare resources (2, 3). Although total hip arthroplasty is an effective treatment for end-stage SONFH, pharmacological strategies that preserve the native joint are still urgently needed for early- to mid-stage patients, for whom current hip-preserving options, including low molecular weight heparin, bisphosphonates, and BMSC transplantation, among others, remain controversial in terms of efficacy, indications, and safety (2). Therefore, a positive drug control was not included. However, no specific drug is currently available for SONFH, making the identification of effective therapeutic markers particularly important (4).The pathological essence of SONFH is the disruption of homeostasis in the local microenvironment of bone tissue, involving the interplay of multiple pathological processes such as an imbalance between osteogenic and adipogenic differentiation of bone marrow mesenchymal stem cells (BMSCs), osteocyte apoptosis, microcirculatory disturbance, and inflammatory responses (5–7). Glucocorticoids can inhibit Wnt/β-catenin signaling activity and activate PPARγ, promoting the directed differentiation of BMSCs into adipocytes while concurrently inducing osteoblast apoptosis and osteoclast activation, ultimately leading to a dual imbalance of decreased bone formation and increased bone resorption (8, 9). In addition, hormone-induced oxidative stress injury and vascular endothelial dysfunction are important driving factors in SONFH progression (10). Although the above mechanisms have been partially validated at the cellular and animal levels, previous studies have mostly focused on single signaling pathways or alterations in individual genes/proteins, lacking a systematic understanding of the overall molecular network dysregulation in SONFH.

In recent years, the rapid development of systems biology technologies such as metabolomics and proteomics has provided unprecedented opportunities for studying complex disease mechanisms. Metabolomics can capture the global dynamic changes of endogenous small-molecule metabolites under disease conditions, directly reflecting the final biochemical phenotype of the organism; proteomics, on the other hand, can reveal aberrant activation or inhibition of signaling pathways at the level of proteins, the functional executors (11, 12). The combined application of these two approaches facilitates the construction of a multi-level regulatory network of “metabolite–protein–signaling pathway, “ compensating for the insufficient information dimension of single-omics approaches. Currently, metabolomics and proteomics have been successfully applied in studies of bone metabolic diseases such as osteoporosis and osteoarthritis, but integrated multi-omics studies in the SONFH field remain very limited (13, 14).However, previous single-omics studies have mainly described individual metabolic or protein alterations and cannot determine whether these changes are functionally coupled. Metabolomics reflects biochemical phenotypes, whereas proteomics reveals protein-level regulation; their integration is therefore needed to link metabolites to protein networks. We hypothesized that Tanshinone IIA may protect against SONFH by modulating this metabolic–protein network, and asked which pathways and candidate nodes are altered and whether Tanshinone IIA reverses them.

Tanshinone IIA is a liposoluble active component extracted from the traditional Chinese medicine Salvia miltiorrhiza Bunge, belonging to the tanshinone monomers. It possesses various pharmacological activities including anti-inflammatory, antioxidant, anti-apoptotic, and pro-angiogenic effects (15–17). Recently, research on the bone-protective effects of Tanshinone IIA has gradually attracted attention. *In vitro* studies have confirmed that Tanshinone IIA can promote the osteogenic differentiation of BMSCs by activating BMP/Smad and Wnt/β-catenin signaling pathways while inhibiting RANKL-induced osteoclastogenesis; animal experiments have also shown that Tanshinone IIA can ameliorate glucocorticoid-induced bone microarchitecture deterioration and bone loss (18–20). However, systematic mechanistic studies on the prevention and treatment of SONFH with Tanshinone IIA are still relatively scarce, and its multi-target regulatory characteristics need to be systematically evaluated at the omics level.

Based on the above background, this study employed ultra-performance liquid chromatography–tandem mass spectrometry (UPLC-MS/MS)-based untargeted metabolomics combined with data-independent acquisition(DIA) quantitative proteomics. Using a hormone-induced rat model of femoral head necrosis as the research object, we systematically delineated the metabolic and protein expression profiles of SONFH and comprehensively evaluated the interventional effects of Tanshinone IIA. The aim is to reveal the core metabolic disturbance nodes and key signaling pathways of SONFH from a multi-omics perspective and to elucidate the multi-target regulatory network of Tanshinone IIA, thereby providing experimental evidence for understanding the pathogenesis of SONFH and for the clinical translation of Tanshinone IIA.

## 2 Methods

### 2.1 Animal grouping

After one week of adaptive feeding, 24 SPF-grade SD rats were numbered according to body weight. Twenty-four consecutive random numbers were copied from a random number table and recorded below the rat numbers, and the 24 random numbers were sorted from smallest to largest. Rats corresponding to ranks 1–8 were assigned to the Control group, those corresponding to ranks 9–16 to the model group, and those corresponding to ranks 17–24 to the Tanshinone IIA group, with eight rats per group. The animal experimental protocol was reviewed and approved by the Experimental Animal Ethics Committee of Shandong Academy of Chinese Medicine, approval number: SDZYY20201210001.All animal experiments were conducted in accordance with the ARRIVE guidelines 2.0 and the 3R principles.

### 2.2 Main reagents and consumables

BCA protein assay kit (Beyotime Biotechnology, China, Cat. No. P0010), protease inhibitor (Thermo Fisher Scientific, USA, Cat. No. A32955), phosphatase inhibitor (Thermo Fisher Scientific, USA, Cat. No. A32961), iST (96×) (PreOmics GmbH, Germany, Cat. No. PO00027), trypsin (Promega, USA, Cat. No. V5113), SDS-PAGE protein loading buffer (5×) (Beyotime Biotechnology, China, Cat. No. P0015), Protein Ladder (unstained) (Beyotime Biotechnology, China, Cat. No. P0063), Pierce™ Quantitative Colorimetric Peptide Assay (Thermo Fisher Scientific, USA, Cat. No. 23275); methylprednisolone sodium succinate for injection (National Medical Products Administration Approval No. HC20150040, Nang Kuang Pharmaceutical Co., Ltd.);Tanshinone IIA (MedChemExpress, USA, Cat. No. HY-N0135, CAS No. 568-72-9, formula C_19_H_18_O_3_, molecular weight 294.34, purity ≥ 99.7%) universal tissue fixative and EDTA decalcifying solution were purchased from Wuhan Servicebio Technology Co., Ltd.; isoflurane (Shandong Ante Animal Husbandry Technology Co., Ltd., 20221004); micro-CT (PerkinElmer Instruments, Quantum GX2); high-speed centrifuge (Eppendorf, Germany, 5430 R); high-speed low-temperature tissue grinder (Wuhan Servicebio Technology Co., Ltd., KZ-III-FP); microplate reader (Bio-Rad, USA, xMark); biosafety cabinet; ultra-clean workbench (Suzhou Antai Airtech Co., Ltd., SW-CJ-2FD); refrigerator (YC-300L); digital constant-temperature water bath (Shanghai Lichen Bangxi Instrument Technology Co., Ltd., HH-2); autoclave sterilizer (STIK Instrument & Equipment (Shanghai) Co., Ltd., IMJ-54A); electric forced-air drying oven (Shanghai Boxun Industrial Co., Ltd. Medical Equipment Factory, GZX-9140 MBE); ultra-low temperature freezer (Thermo Fisher Scientific (Suzhou), 902GP), etc.

### 2.3 Modeling and intervention

Rats in the model group and the Tanshinone IIA group were injected with methylprednisolone sodium succinate (30 mg·kg^-^¹·d^-^¹) into the right gluteal muscle to establish the SONFH model, administered on the first three consecutive days each week for three weeks (21). Rats in the Control group received an equal dose and frequency of normal saline injection into the right gluteal muscle. Tanshinone IIA (purity ≥ 99.7%) is a pure chemical entity, not a decoction piece or crude extract. Due to its poor aqueous solubility, Tanshinone IIA powder was uniformly suspended in drinking water by vortexing to prepare a working suspension at 0.8 mg/mL. The suspension was prepared fresh daily and vortexed thoroughly immediately before each administration. Each rat was weighed before gavage, and the suspension was administered by oral gavage at 10 mL/kg body weight, corresponding to 8 mg·kg^-^¹·d^-^¹ for six consecutive weeks (22). The control and model groups received an equal volume of drinking water by gavage. No DMSO or other organic solvent was used in the formulation.

### 2.4 Sample collection and processing

After the intervention, rats were fasted with water deprivation for 12 h, anesthetized with isoflurane inhalation, and then sacrificed. Bilateral femoral heads and fecal samples were collected. The right femoral head and fecal samples were snap-frozen in liquid nitrogen and stored at −80 °C for later use; the left femoral head was placed in universal tissue fixative for fixation.All samples were randomly selected and coded with numeric labels before analysis. Histological scoring and Micro-CT analysis were performed in a double-blinded manner by two independent observers who were blinded to group allocation. Metabolomic and proteomic data analyses were also performed by researchers blinded to group identity.

### 2.5 Micro-CT scanning and analysis

Right femoral head samples from three randomly selected rats in each group were scanned using a Micro-CT scanner, and three-dimensional reconstruction was performed using the micro-tomography imaging system to observe the radiographic morphological changes of the femoral head. Analyze 12.0 software was used to calculate bone mean (B.M), trabecular bone volume (BV), tissue volume (TV), trabecular thickness (Tb.TH), and trabecular separation (Tb.Sp). The bone volume fraction (BV/TV) was subsequently computed.

### 2.6 Histopathological observation of the femoral head

The fixed left femoral heads were rinsed with PBS and decalcified in 5% EDTA solution for 4–6 weeks, with the decalcification solution changed daily until a needle tip could be easily inserted. After decalcification, gradient dehydration, paraffin embedding, and serial sectioning at 5 μm thickness were performed. HE staining: following the kit instructions, sections were deparaffinized to water, stained with hematoxylin, differentiated in hydrochloric acid alcohol, blued, stained with eosin, dehydrated and cleared, and mounted with neutral gum. The arrangement, density, continuity of bone trabeculae, and adipocyte infiltration in the marrow cavity were observed under a light microscope. TRAP staining: tartrate-resistant acid phosphatase staining was used. Osteoclast positive staining was observed under a light microscope, and the number and activity of osteoclasts in each group were counted and compared.

### 2.7 Untargeted metabolomics analysis

Fecal samples from six rats per group were sent to Guangzhou Gidio Biotechnology Co., Ltd. for untargeted metabolomics detection. Fecal samples were chosen because they are non-invasive and reflect systemic metabolic status. Metabolite separation and detection were performed using an Agilent 1290 Infinity LC ultra-high performance liquid chromatography system. Metabolite data were normalized and standardized using the Omicsmart analysis platform. Orthogonal partial least squares discriminant analysis (OPLS-DA) models were employed to analyze the differences in metabolites among groups, and permutation tests were used to verify model reliability. Differential metabolites were screened using the criteria of |log_2_FC| > 1, FDR P-value ≤ 0.05, and variable importance in projection (VIP) ≥ 1. The differential metabolites were mapped to the KEGG database for pathway enrichment analysis.

### 2.8 Proteomics analysis

Femoral head samples from five rats per group were sent to Guangzhou Gidio Biotechnology Co., Ltd. for proteomic sequencing. These five rats were matched with the metabolomics samples used for correlation analysis. Proteomic data were normalized and standardized using the Omicsmart analysis platform. Principal component analysis (PCA) models were used to analyze the differences in protein expression among groups. Differentially expressed proteins were screened using the criteria of |log_2_FC| > 1 and FDR < 0.05 and were mapped to the KEGG database for pathway enrichment analysis. Pearson correlation analysis was used to assess the correlations between differential proteins and differential metabolites. The P values from Pearson correlation analysis were adjusted for multiple testing using the Benjamini–Hochberg FDR method. Proteins with FDR < 0.05 were considered significantly correlated with differential metabolites. **Figure 1** shows the overall flow chart of the study. Network centrality was not calculated in this study. Candidate proteins were defined as differentially expressed proteins that were significantly correlated with differential metabolites (FDR < 0.05) and supported by literature evidence for roles in bone metabolism, angiogenesis, oxidative stress, or inflammation.

**Figure 1:**
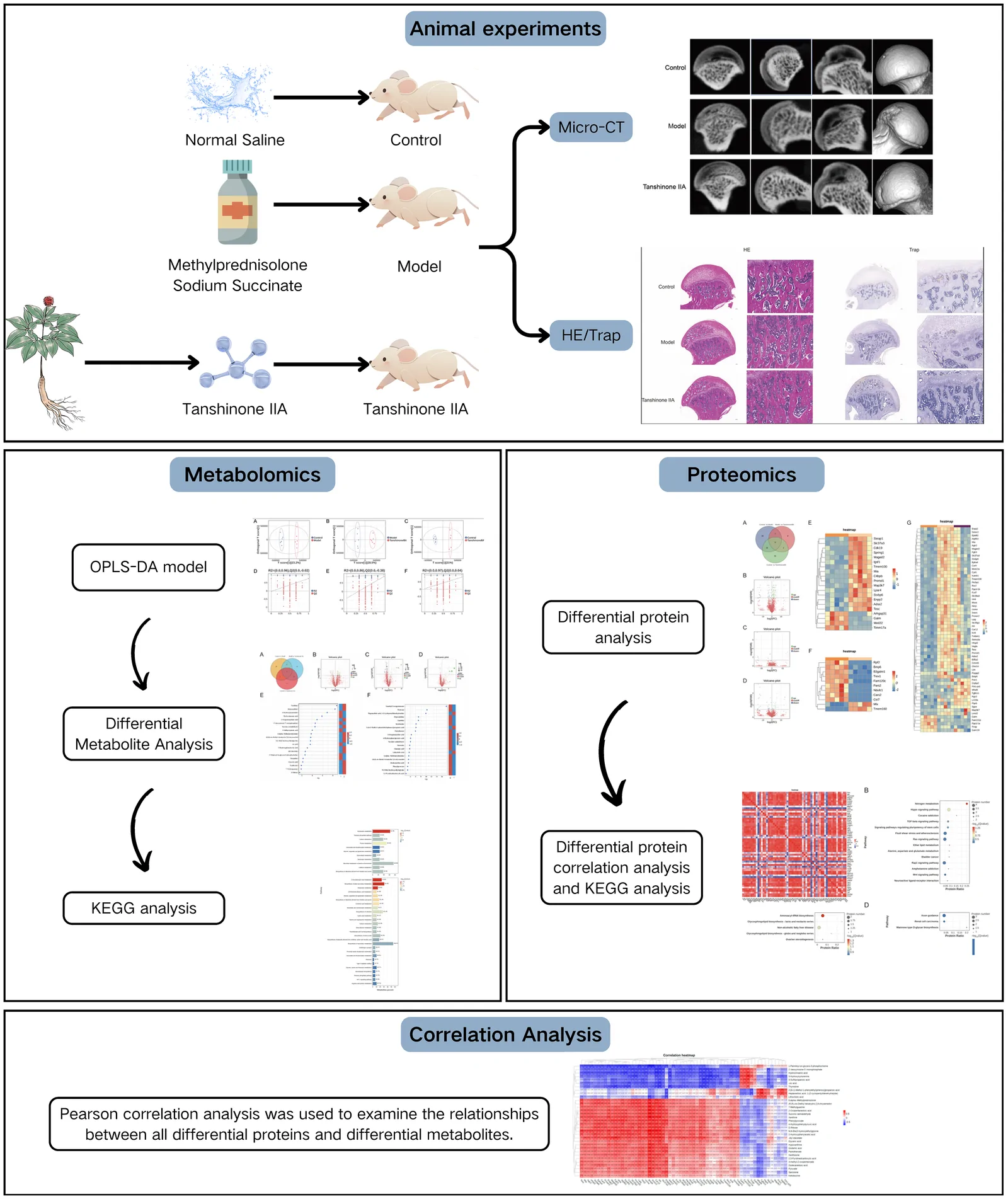
Flow chart of the study.

### 2.9 Statistical analysis

Data are presented as mean ± SD. For Micro-CT and histological quantification, comparisons among the three groups were performed using one-way ANOVA followed by Tukey’s *post hoc* test, and P < 0.05 was considered statistically significant. For metabolomic and proteomic differential analyses, as well as Pearson correlation analysis, P values were adjusted using the FDR method, and FDR < 0.05 was considered statistically significant. All statistical analyses were performed using GraphPad Prism (version 10.1.2, GraphPad Software, USA).

## 3 Results

### 3.1 Femoral head Micro-CT and pathological staining results

Micro-CT three-dimensional reconstruction showed (**Figure 2A**) that the femoral heads of rats in the Control group had regular contours, orderly trabecular arrangement, and no cystic degeneration; the model group showed irregular femoral head contours, marked loss and disorganization of trabeculae, and partial cystic degeneration. Quantitative analysis indicated that compared with the Control group, the model group had significantly decreased bone volume and bone volume fraction and increased tissue volume, suggesting bone loss and medullary cavity expansion. Compared with the model group, the Tanshinone IIA group showed markedly recovered bone volume and bone volume fraction, reduced tissue volume, significantly improved bone microarchitecture destruction, and a tendency toward orderly trabecular arrangement (**Figure 2B**). These results suggest that Tanshinone IIA may ameliorate the bone microarchitecture degeneration caused by femoral head necrosis.

**Figure 2:**
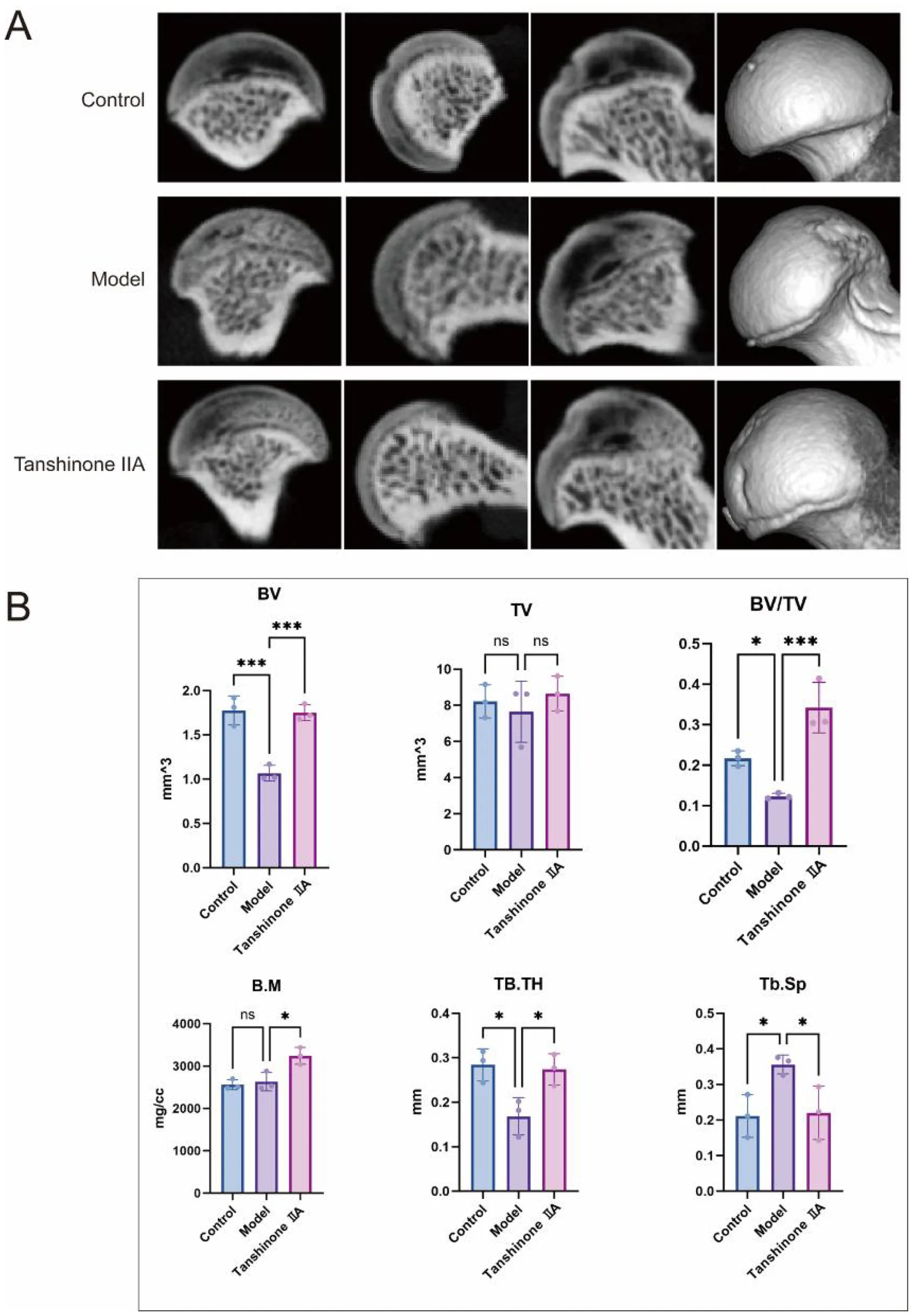
Micro-CT evaluation of femoral head bone microarchitecture changes in each group of rats.(n=3). **(A)** Three-dimensional reconstructed images of femoral heads from each group, showing trabecular morphology and distribution. **(B)** Comparison of quantitative bone microarchitecture indices among groups, including bone mean (B.M), bone volume (BV), bone volume fraction (BV/TV), Trabecular Thickness(TB.TH), Trabecular Separation(TB.Sp), tissue volume (TV); ns indicates no statistical difference.

### 3.2 Pathological staining results

HE staining in **Figures 3A, C** showed that the Control group had uniformly distributed bone trabeculae without adipocyte infiltration; the model group had sparse or interrupted trabeculae with evident adipocyte infiltration; the Tanshinone IIA group exhibited denser trabeculae than the model group and reduced adipocyte infiltration. TRAP staining in **Figures 3B, D** showed that osteoclasts were scarce in the Control group, with only scattered positive staining; the model group showed a significantly increased number of osteoclasts, clustered focally with intensified staining; the Tanshinone IIA group had a markedly reduced number of osteoclasts and decreased staining intensity compared with the model group.

**Figure 3:**
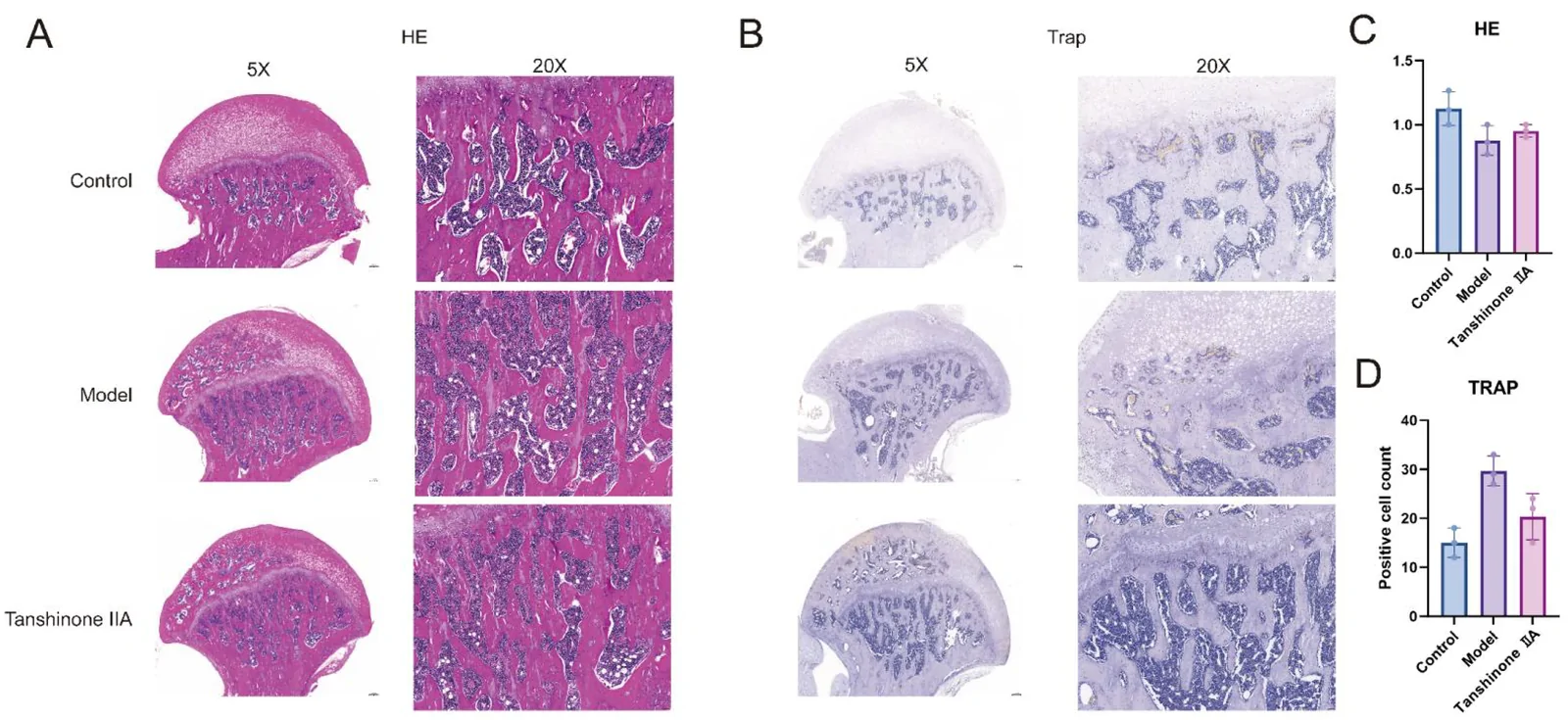
Histological staining observation of femoral heads in each group of rats.(n=3). **(A)** HE staining to observe trabecular morphology and marrow cavity structure of femoral heads in each group (5× and 20× magnification). **(B)** TRAP staining to evaluate osteoclast activity in each group (5× and 20× magnification). **(C)** Quantitative analysis of trabecular area in HE staining (normalized to the Control group). **(D)** Quantitative analysis of TRAP-positive cell count.

### 3.3 Metabolite detection and analysis results

#### 3.3.1 OPLS-DA model analysis results

OPLS-DA was applied for pattern recognition of the metabolite profiles of the three groups. The OPLSDA score scatter plots for pairwise group comparisons are shown in **Figures 4A–C**. Permutation-test-derived intercept values for R²Y and Q² were calculated for each pairwise OPLS-DA model (**Figures 4D–F**). The intercepts were: Control-vs-Model R²Y = 0.96, Q² = -0.02; Model-vs-Tanshinone IIA R²Y = 0.86, Q²= -0.38; Control-vs-Tanshinone IIA R²Y = 0.97, Q²= 0.04. Permutation tests demonstrated no obvious over-fitting for all three models. Notably, the Control-versus-Tanshinone IIA comparison yielded a low positive Q²=0.04. While this value excludes serious over-fitting, it also indicates that global fecal metabolomic differences between the control and Tanshinone IIA groups are modest. This observation supports our description of partial restoration of metabolic homeostasis (**Figure 4**).

**Figure 4:**
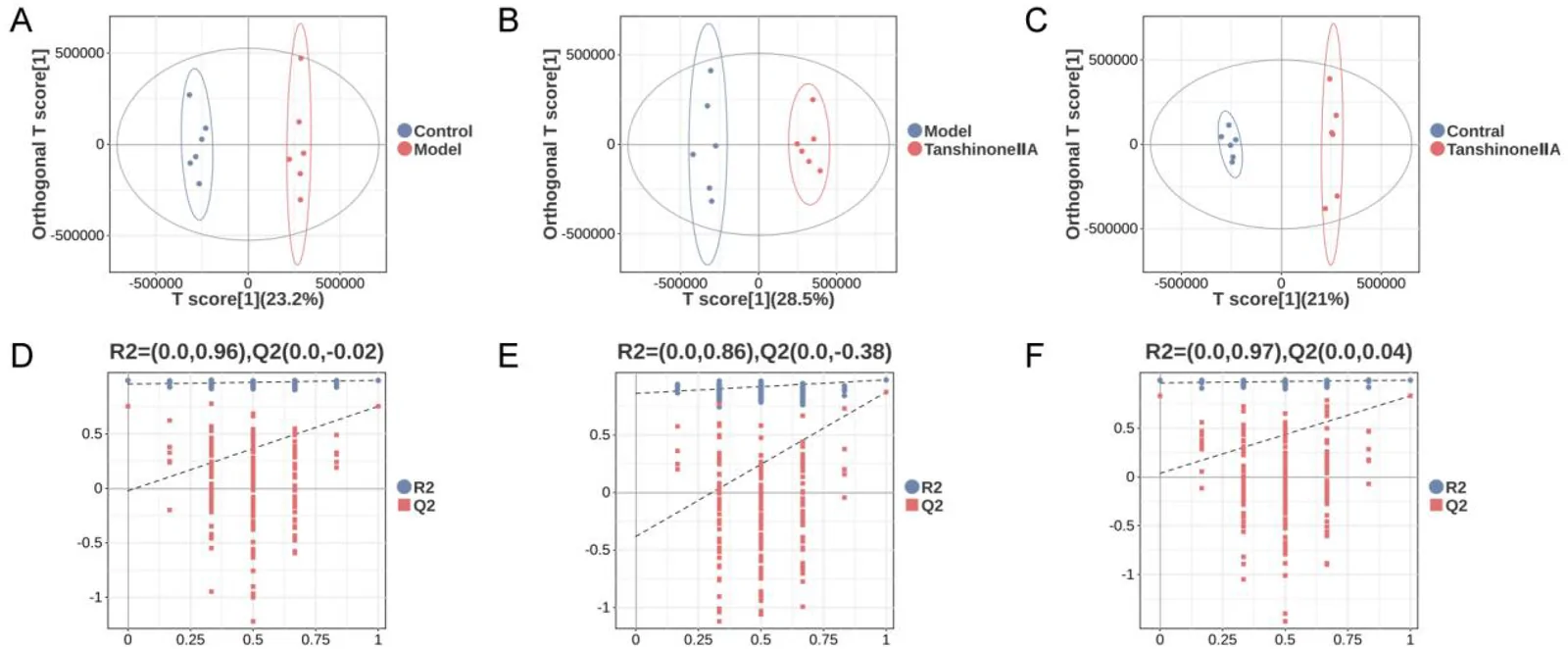
OPLS_DA score scatter plots and permutation test results for pairwise comparison. **(A)** Score scatter plot of Control versus Model group; **(B)** Score scatter plot of Model versus Tanshinone IIA group; **(C)** Score scatter plot of Control versus Tanshinone IIA group. **(D–F)** Permutation test results of the corresponding OPLS_DA models for panels **(A–C)** respectively. R²Y (blue) and Q² (red) intercept values obtained from permutation testing are annotated for each pairwise comparison (n=6).

#### 3.3.2 Differential metabolite analysis results

UPLC-MS/MS-based untargeted metabolomics was used to screen differential metabolites (VIP > 1, |log_2_FC| > 1, FDR < 0.05) (**Figures 5A–D**). The VIP-score-ranked top-20 metabolites were further characterized to interpret metabolic perturbations (**Figures 5E, F**). Compared with the control group, 18 differential metabolites were screened in the model group. 11 metabolites such as xanthine, and 2-oxopentanedioic acid were significantly upregulated, while 7 metabolites including 3-hydroxykynurenine and hydrocinnamic acid were downregulated (**Table 1**). VIP scores revealed that xanthine and Hypoxanthine were the top contributors, suggesting marked disruption of purine metabolism and altered organic acid metabolism in the model animals (**Figure 5E**).

**Figure 5:**
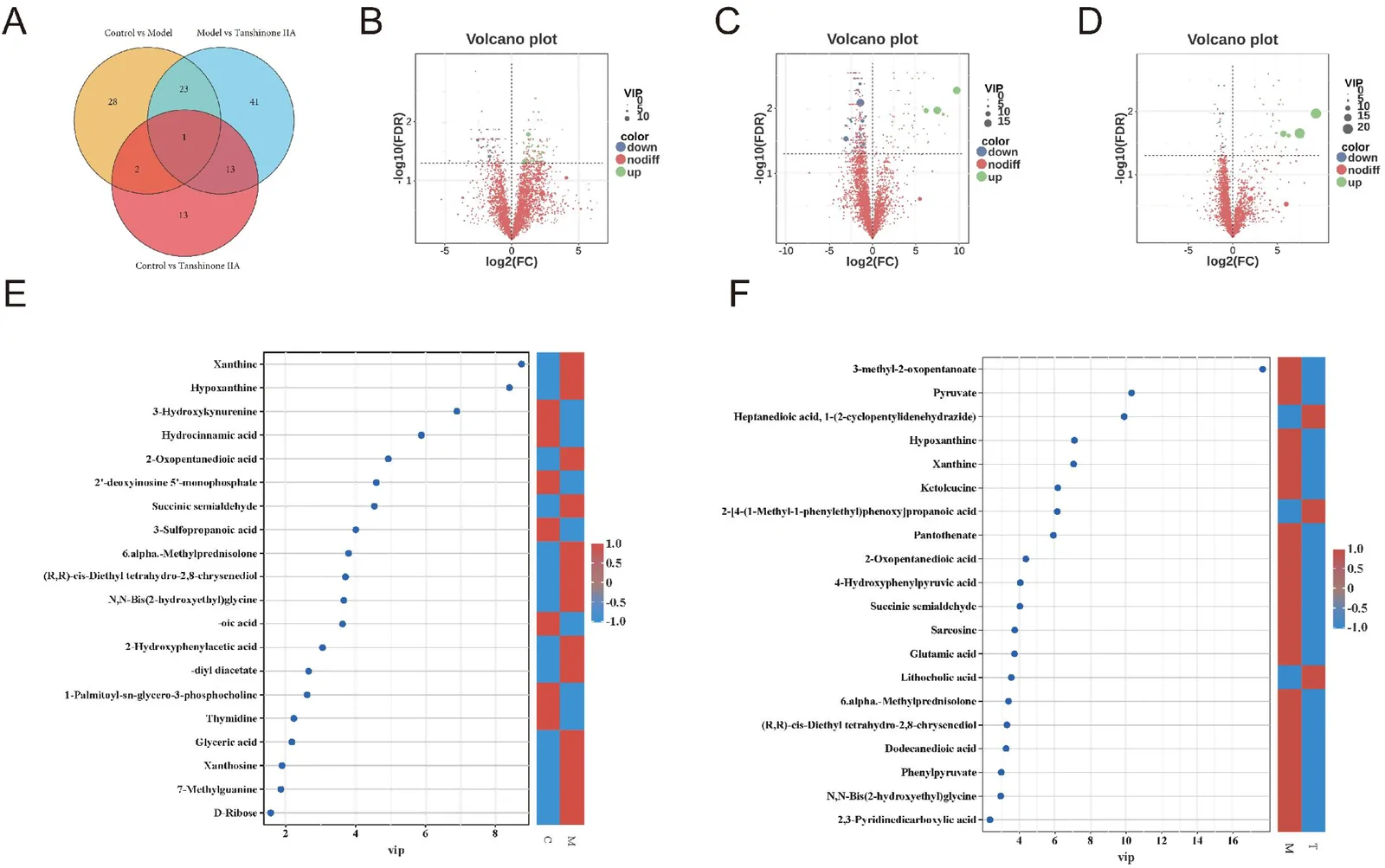
Multi-dimensional analysis of differential metabolites based on UPLC-MS/MS. **(A)** Venn diagram of differential metabolites among the three groups, showing the number of overlapping and unique differential metabolites in each comparison. **(B–D)** Volcano plots of differential metabolites for each pairwise comparison [**(B)** Control vs Model; **(C)** Model vs Tanshinone IIA; **(D)** Control vs Tanshinone IIA]. **(E, F)** VIP score plots of key differential metabolites [**(E)** Control vs Model; **(F)** Model vs Tanshinone IIA], where higher VIP values indicate a greater contribution to group discrimination.

**Table 1:** Identification of metabolites in feces between the control and model groups(top-20 metabolites ranked by VIP score).

Name	Class	log_2_ (FC)	*FDR-P*	VIP	m/z
Xanthine	Imidazopyrimidines	1.241	0.016	8.748	151.026
Hypoxanthine	Imidazopyrimidines	0.951	0.047	8.397	137.046
3-Hydroxykynurenine	Organooxygen_compounds	-1.664	0.039	6.896	206.046
Hydrocinnamic acid	Phenylpropanoic_acids	-2.541	0.020	5.880	149.061
2-Oxopentanedioic acid	Keto_acids_and_derivatives	2.289	0.033	4.932	145.014
2’-deoxyinosine 5’-monophosphate	Purine_nucleotides	-1.438	0.020	4.590	331.249
Succinic semialdehyde	Fatty_Acyls	2.173	0.036	4.537	101.024
3-Sulfopropanoic acid	Organic_sulfonic_acids_and_derivatives	-2.105	0.020	4.004	152.986
6.alpha.-Methylprednisolone	Steroids_and_steroid_derivatives	1.343	0.020	3.801	321.181
(R, R)-cis-Diethyl tetrahydro-2, 8-chrysenediol	Phenanthrenes_and_derivatives	1.313	0.027	3.709	319.166
N, N-Bis(2-hydroxyethyl)glycine	Carboxylic_acids_and_derivatives	2.015	0.007	3.663	164.093
-oic acid	Steroids_and_steroid_derivatives	-2.274	0.027	3.625	467.278
2-Hydroxyphenylacetic acid	Benzene_and_substituted_derivatives	2.641	0.027	3.054	151.040
-diyl diacetate	Oxepanes	2.660	0.021	2.651	589.302
1-Palmitoyl-sn-glycero-3-phosphocholine	Glycerophospholipids	-1.612	0.031	2.605	496.339
Thymidine	Pyrimidine_nucleosides	-1.385	0.027	2.230	241.083
Glyceric acid	Organooxygen_compounds	1.276	0.048	2.175	105.019
Xanthosine	Purine_nucleosides	1.091	0.028	1.893	283.068
7-Methylguanine	Imidazopyrimidines	0.904	0.023	1.860	166.072
D-Ribose	Organooxygen_compounds	1.097	0.041	1.570	149.046

After Tanshinone IIA intervention, 19 differential metabolites were screened between the model group and Tanshinone IIA group (**Table 2**). 3 metabolites were increased after treatment, while 16 metabolites were decreased, such as pyruvate, hypoxanthine and xanthine.

**Table 2:** Identification of metabolites in feces between the model and Tanshinone IIA groups (top-20 metabolites ranked by VIP score).

Name	Class	log_2_ (FC)	*FDR-* *P*	VIP	m/z
3-methyl-2-oxopentanoate	Keto_acids_and_derivatives	-1.408	0.008	17.681	129.056
Pyruvate	Keto_acids_and_derivatives	-3.075	0.029	10.31	87.009
Heptanedioic acid, 1-(2-cyclopentylidenehydrazide)	Fatty_Acyls	6.185	0.011	9.896	239.144
Hypoxanthine	Imidazopyrimidines	-1.027	0.035	7.095	137.046
Xanthine	Imidazopyrimidines	-1.165	0.016	7.052	151.026
Ketoleucine	Keto_acids_and_derivatives	-1.449	0.004	6.164	129.056
2-[4-(1-Methyl-1-phenylethyl)phenoxy]propanoic acid	Benzene_and_substituted_derivatives	8.172	0.012	6.140	283.134
Pantothenate	Organooxygen_compounds	-1.319	0.038	5.916	218.104
2-Oxopentanedioic acid	Keto_acids_and_derivatives	-2.565	0.015	4.375	145.014
4-Hydroxyphenylpyruvic acid	Benzene_and_substituted_derivatives	-3.109	0.018	4.055	179.035
Succinic semialdehyde	Fatty_Acyls	-2.447	0.016	4.035	101.024
Sarcosine	Carboxylic_acids_and_derivatives	-1.481	0.034	3.747	88.040
Glutamic acid	Carboxylic_acids_and_derivatives	-1.409	0.026	3.734	146.046
Lithocholic acid	Steroids_and_steroid_derivatives	2.271	0.016	3.545	375.290
6.alpha.-Methylprednisolone	Steroids_and_steroid_derivatives	-1.462	0.013	3.395	321.181
(R, R)-cis-Diethyl tetrahydro-2, 8-chrysenediol	Phenanthrenes_and_derivatives	-1.415	0.020	3.308	319.166
Dodecanedioic acid	Fatty_Acyls	-1.405	0.031	3.262	229.145
Phenylpyruvate	Benzene_and_substituted_derivatives	-2.362	0.045	2.99	163.04
N, N-Bis(2-hydroxyethyl)glycine	Carboxylic_acids_and_derivatives	-1.783	0.005	2.956	164.093
2, 3-Pyridinedicarboxylic acid	Pyridines_and_derivatives	-0.845	0.031	2.348	122.025

*a* The abbreviated names “-oic acid” and “-diyl diacetate” in this table correspond to the following full chemical names, respectively: (17Z)-16-(Acetyloxy)-6-hydroxy-4, 8, 14-trimethyl-3, 7-dioxo-18-norcholesta-17, 24-dien-21-oic acid, and 7-Hydroxy-10-(2-hydroxypropan-2-yl)-2, 2a, 4, 7b, 13a-pentamethyl-6-oxo-2a, 3, 4, 6, 7, 7a, 7b, 8, 9, 10, 11a, 12, 13, 13a-tetradecahydro-2H-oxeto[2′, 3′:1, 5]cyclopenta[1, 2-h]pyrano[3, 2-a]xanthene-3, 8-diyl diacetate. The same abbreviations are used consistently throughout all subsequent figures and tables.

Notably, xanthine, Hypoxanthine, 2-oxopentanedioic acid, succinic semialdehyde, and N, N-bis(2-hydroxyethyl)glycine increased in the model group and markedly declined after Tanshinone IIA intervention. In contrast, pyruvate and glutamic acid were further decreased in the Tanshinone IIA group compared with the model group. These reductions may reflect increased utilization of these metabolites rather than aggravated metabolic disturbance. The Venn diagram identified these five metabolites as core shared perturbation nodes across the two comparisons. VIP plots (**Figures 5E, F**) confirmed that the metabolic phenotype of the Tanshinone IIA group partially recovered toward the control group, involving purine metabolism, the TCA cycle, and amino acid pathways. Tanshinone IIA partially reversed major metabolic abnormalities in model animals; recovery of purine metabolites and organic acids was most remarkable, offering metabolomic support for its protective action.

Of note, 6α-methylprednisolone was significantly reduced in the Tanshinone IIA group despite identical glucocorticoid administration in both model and Tanshinone IIA groups. We speculate that Tanshinone IIA may alter gut-mediated metabolism or excretion of glucocorticoid metabolites. However, the exact underlying mechanism remains unclear and requires further experimental verification.

#### 3.3.3 KEGG pathway enrichment analysis of differential metabolic pathways

The differential metabolites identified from each comparison were subjected to KEGG pathway enrichment analysis, and the results are shown in **Figure 6**. In the comparison between the control and model groups, the significantly enriched pathways (−log10(Q-value) > 1.0) included nucleotide metabolism and purine metabolism. Combined with the VIP analysis results (xanthine and hypoxanthine) mentioned above, this suggests that the purine metabolism disturbance in the model animals was more manifested as drastic abnormalities of key metabolic nodes rather than a systematic and widespread dysregulation of the pathway.

**Figure 6:**
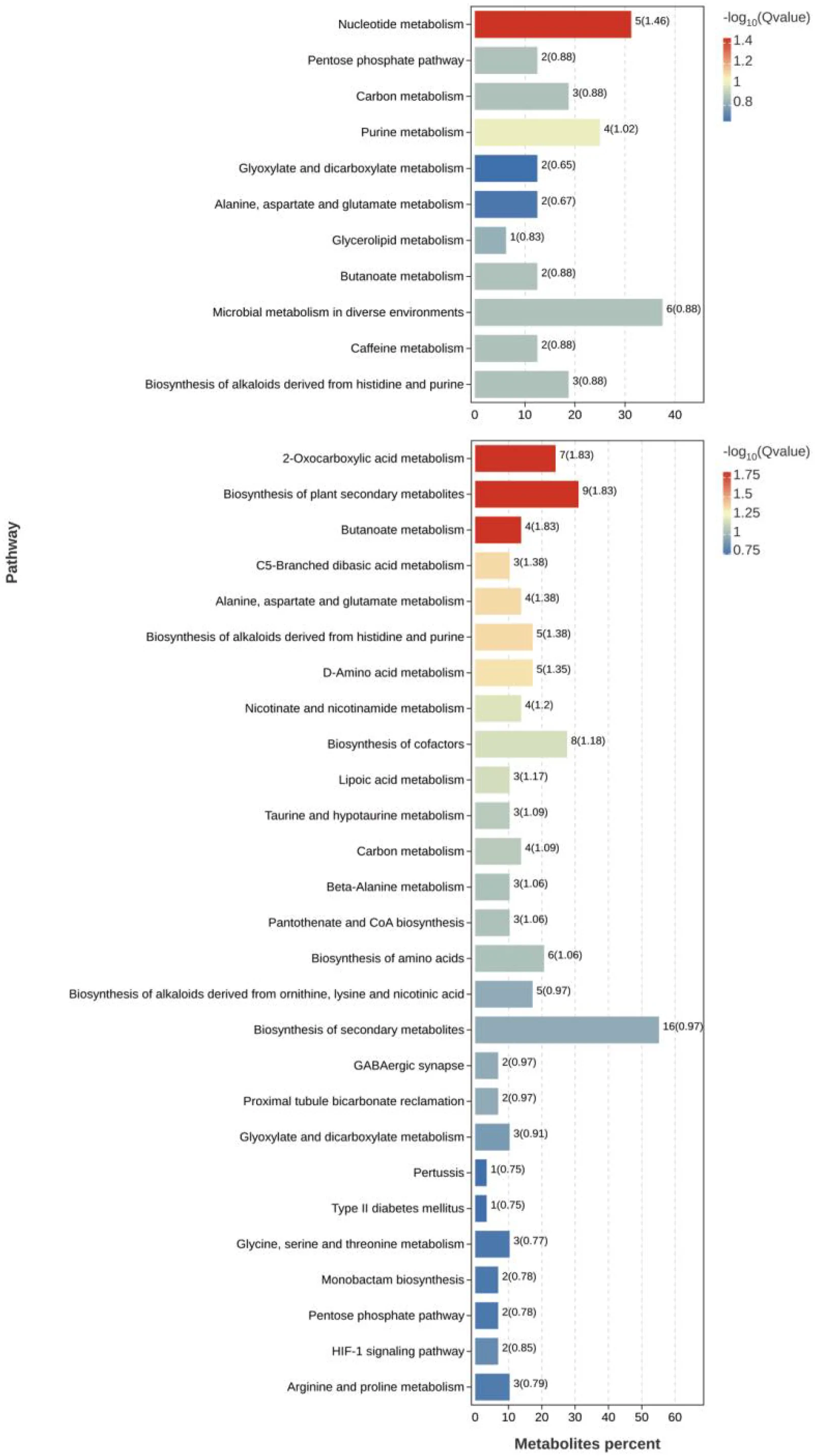
KEGG pathway enrichment analysis of differential metabolites. The ordinate represents the names of enriched KEGG metabolic pathways, and the abscissa represents the enrichment significance (−log10(Q-value)), with larger values indicating more significant enrichment. Bubble size represents the number of differential metabolites enriched in the pathway. Upper panel: control vs. model; lower panel: model vs. Tanshinone IIA. Q-value < 0.05 is considered significantly enriched.

In the comparison between the model and Tanshinone IIA groups, the number of enriched pathways significantly increased and the overall enrichment degree was higher. The most significantly enriched pathways included 2-oxocarboxylic acid metabolism, butanoate metabolism, C5-branched dibasic acid metabolism, alanine, aspartate and glutamate metabolism, and D-amino acid metabolism. In addition, pathways such as carbon metabolism, pantothenate and CoA biosynthesis, and biosynthesis of amino acids also showed enrichment trends. Some pathways, such as biosynthesis of plant secondary metabolites, may reflect KEGG database annotation overlap and should be interpreted with caution. Taken together, the pathways associated with Tanshinone IIA intervention were distributed in amino acid metabolism, energy metabolism, and cofactor synthesis, which is consistent with the finding that key metabolites were significantly restored. These results suggest that Tanshinone IIA may be associated with improved metabolic homeostasis in model animals, with 2-oxocarboxylic acid metabolism as one of the potential links.

### 3.4 Proteomics

Data-independent acquisition (DIA) quantitative proteomics was used to screen differentially expressed proteins (DEPs) in the femoral head samples of the three groups, with screening criteria of |log_2_FC| > 1 and FDR < 0.05. As shown in **Figure 7A**, the Venn diagram displays the distribution of DEPs among the three groups; the volcano plots and clustering heatmaps show the DEP expression characteristics for each comparison and the overall clustering trends of the three groups of samples, respectively. In the comparison between the control and model groups (**Figure 7B**), 116 DEPs were identified, whereas in the comparison between the model and Tanshinone IIA groups (**Figure 7C**), 12 DEPs were identified, among which 3 were significantly upregulated and 9 were significantly downregulated after Tanshinone IIA intervention. The clustering heatmaps (**Figures 7E–G**) showed a clear separation trend of protein expression profiles between the model group and both the control and Tanshinone IIA groups, with the overall protein expression profile of the Tanshinone IIA group regressing toward that of the control group.

**Figure 7:**
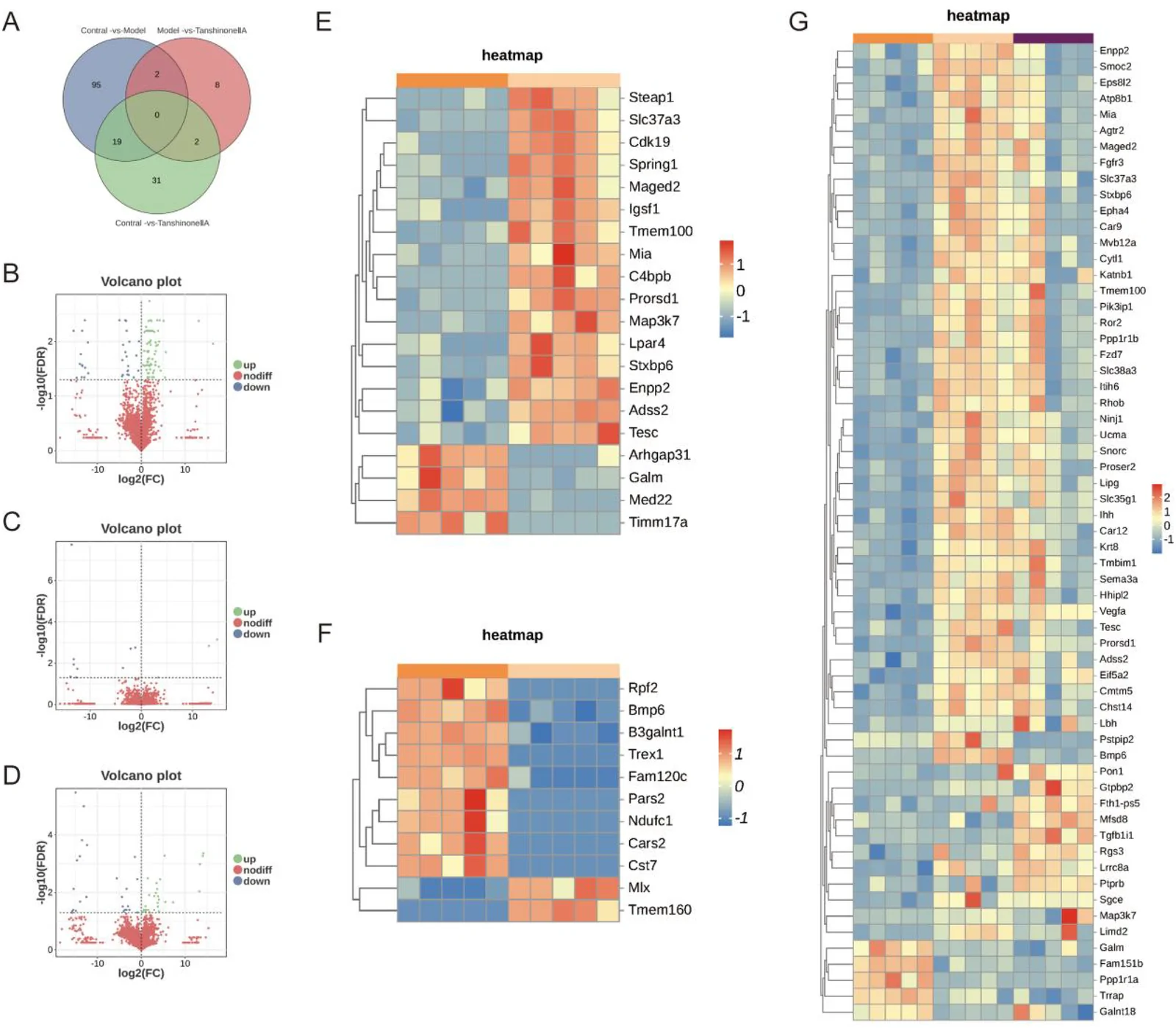
Proteomic differential analysis results. **(A)** Venn diagram of differentially expressed proteins (DEPs) among the control, model, and Tanshinone IIA groups. Numbers indicate the number of DEPs screened in each comparison group (|log_2_FC| > 1, FDR < 0.05), and overlapping areas represent DEPs shared among groups. **(B–D)** Volcano plots of DEPs for control vs. model, model vs. Tanshinone IIA, and control vs. Tanshinone IIA, respectively. The abscissa represents log_2_FC, the ordinate represents −log10(FDR), and red and blue represent significantly upregulated and downregulated proteins, respectively. **(E)** Hierarchical clustering heatmap of DEPs for control vs. model, **(F)** model vs. Tanshinone IIA, with the control and model groups from left to right. **(G)** Hierarchical clustering heatmap of DEPs for the three groups, with the control, model, and Tanshinone IIA groups from left to right. Red and blue indicate upregulation and downregulation of protein expression levels, respectively.

### 3.5 Target protein KEGG analysis

Correlation analysis (**Figure 8A**) showed the degree of correlation of DEP expression levels among the three groups, and the sample clustering was generally consistent with the experimental grouping, indicating good intra-group biological reproducibility. In the control vs. model comparison (**Figure 8B**), significantly enriched pathways included nitrogen metabolism, Hippo signaling pathway, TGF-beta signaling pathway, Ras signaling pathway, Wnt signaling pathway, and neuroactive ligand-receptor interaction, among which nitrogen metabolism was the most significantly enriched. In the model vs. Tanshinone IIA comparison (**Figure 8C**), aminoacyl-tRNA biosynthesis was the most significantly enriched, followed by non-alcoholic fatty liver disease and glycosphingolipid biosynthesis - lacto and neolacto series, suggesting that Tanshinone IIA intervention mainly affected pathways related to aminoacyl-tRNA synthesis and lipid metabolism. In the control vs. Tanshinone IIA comparison (**Figure 8D**), axon guidance was the most significantly enriched, followed by mannose type O-glycan biosynthesis and renal cell carcinoma, indicating that the organism still retained some protein expression characteristics distinct from the control after Tanshinone IIA intervention, which may be related to its sustained pharmacological effects. Collectively, the proteomic KEGG enrichment analysis mutually corroborated the previous metabolomic pathway analysis, jointly revealing that Tanshinone IIA may exert protective effects by regulating nitrogen metabolism, aminoacyl-tRNA biosynthesis, mannose type O-glycan biosynthesis, and lipid metabolism-related pathways.

**Figure 8:**
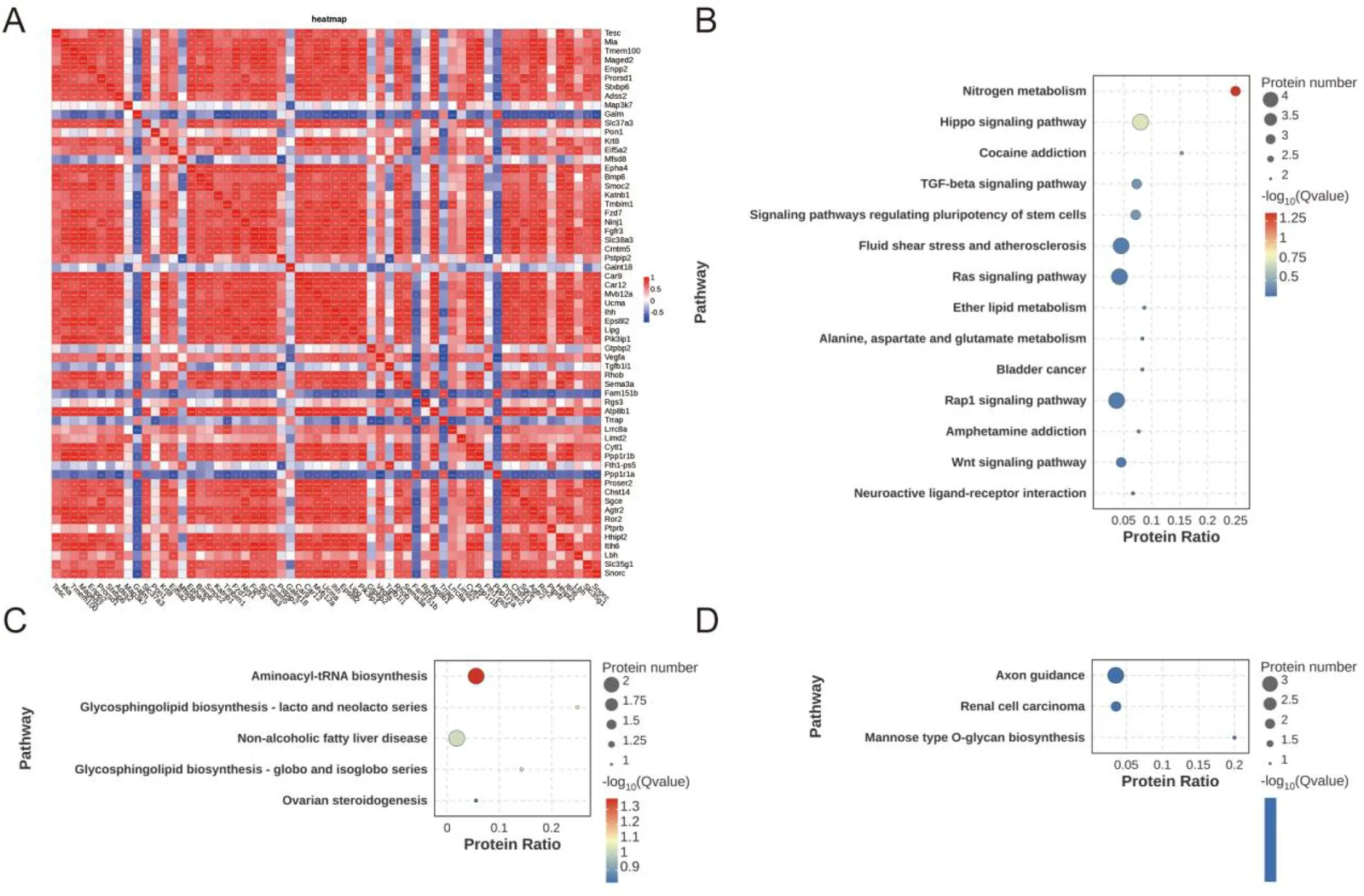
Correlation analysis and KEGG analysis results. **(A)** Heatmap of correlation analysis of differential proteins. **(B–D)** KEGG analysis results for control vs. model, model vs. Tanshinone IIA, and control vs. Tanshinone IIA, respectively.

### 3.6 Correlation analysis

Pearson correlation analysis was performed using data from the five rats per group for which both fecal metabolomic and femoral head proteomic data were available, ensuring matched individual animals, and the results are shown in **Figure 9**. After Benjamini–Hochberg FDR correction, the analysis revealed that Bmp6, Smoc2, Ihh, and Enpp2 had significant correlations with differential metabolites (FDR<0.05). These results indicate that these proteins may serve as key protein nodes linking purine metabolism, energy metabolism, and lipid metabolism disorders, and they may coordinately participate in the pathological process of the model animals and the interventional effects of Tanshinone IIA together with the differential metabolites. This provides direct multi-omics evidence for elucidating the multi-target regulatory mechanism of Tanshinone IIA.

**Figure 9:**
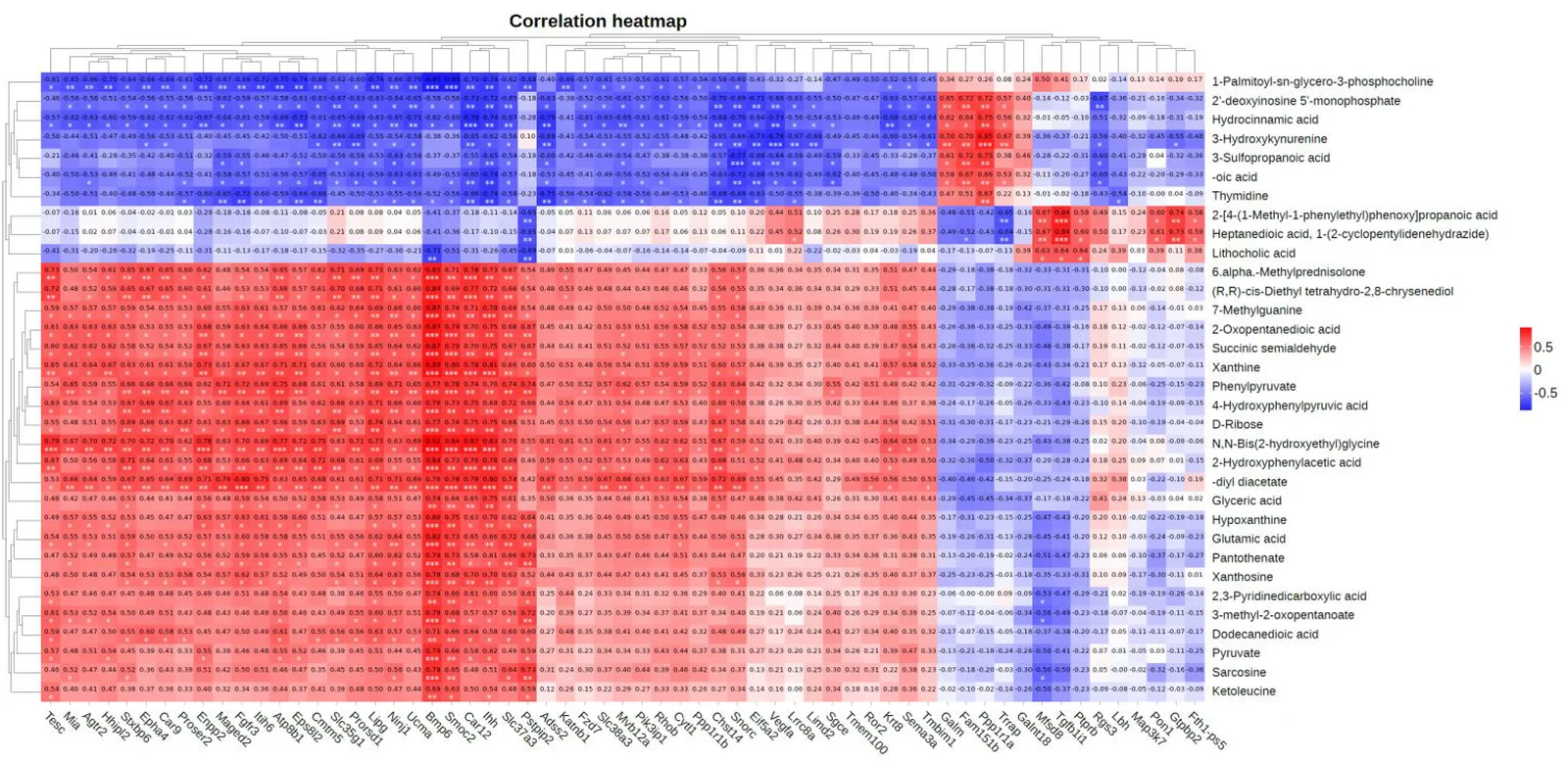
Correlation analysis was based on matched fecal metabolomic and femoral head proteomic data from the same five rats per group (metabolomics: n = 6 per group; proteomics: n = 5 per group; correlation analysis: n = 5 per group with both datasets available). Pearson correlation P values were adjusted using the Benjamini–Hochberg FDR method.

## 4 Discussion

In this study, we integrated Micro-CT, histopathology, untargeted metabolomics, and DIA proteomics to systematically evaluate the interventional effects of Tanshinone IIA on a rat model of SONFH. The multi-omics data demonstrate that Tanshinone IIA not only ameliorates femoral head bone microarchitecture destruction and fatty infiltration but also exerts systematic regulatory effects at the metabolic network and protein expression levels, suggesting that it may delay the progression of SONFH through a multi-target synergistic mechanism.

The core pathological mechanism of SONFH involves multiple processes including an imbalance between adipogenic and osteogenic differentiation of BMSCs, microvascular endothelial injury, abnormalities in the coagulation-fibrinolysis system, and osteocyte apoptosis (23). Excess glucocorticoids can activate peroxisome proliferator-activated receptor γ (PPARγ) and CCAAT/enhancer-binding protein α (C/EBPα), driving the differentiation of BMSCs into adipocytes, while simultaneously inhibiting the Wnt/β-catenin signaling pathway and weakening osteogenic differentiation capacity. This leads to sparse trabeculae, massive accumulation of adipocytes in the marrow cavity, and intraosseous hypertension, further compressing microvessels and causing ischemic necrosis (24). The sparse and interrupted trabeculae with evident adipocyte infiltration observed in the model group by HE staining in this study are highly consistent with the above pathological mechanism. After Tanshinone IIA intervention, the trabeculae tended to be denser and fatty infiltration was alleviated, suggesting that it may effectively reverse the hormone-induced abnormal differentiation of BMSCs. The literature confirms that Tanshinone IIA can promote the osteogenic differentiation of BMSCs by activating Wnt/β-catenin signaling while inhibiting adipogenic differentiation by downregulating the expression of PPARγ and C/EBPα (25). Recent studies further indicate that Tanshinone IIA promotes BMSC osteogenesis and inhibits adipogenesis by regulating the TGFβ signaling pathway, providing direct mechanistic support for explaining the improved bone microarchitecture and reduced fatty infiltration observed in this study (18). In addition, Tanshinone IIA can reduce osteoclastogenesis by inhibiting RANKL-induced activation of the NF-κB and MAPK pathways (20). The reduction in osteoclasts shown by TRAP staining in this study is consistent with this, suggesting that the bone destruction in SONFH also involves enhanced osteoclast activity, which is inhibited by Tanshinone IIA.

Metabolomics revealed a significant accumulation of purine metabolites (xanthine, hypoxanthine) and abnormal elevation of 2-oxopentanedioic acid (a TCA cycle-related metabolite) and succinic semialdehyde (an intermediate of the GABA shunt/4-aminobutanoate metabolism) in the model animals. After Tanshinone IIA intervention, these metabolites all returned toward normal levels. In the pathological context of SONFH, the accumulation of purine metabolites is not merely a metabolic disturbance but a direct biochemical marker of ischemia and hypoxia (26). After interruption of the local blood supply to the femoral head, intracellular ATP synthesis is blocked and ATP is largely degraded to ADP and AMP, which are subsequently broken down by 5’-nucleotidase and purine nucleoside phosphorylase to hypoxanthine and xanthine. These are finally catalyzed by xanthine oxidase to produce uric acid accompanied by a large amount of reactive oxygen species (ROS) (27). Studies have shown that glucocorticoids can upregulate xanthine oxidase activity, exacerbating oxidative stress damage to vascular endothelial cells and promoting microthrombus formation (28). Tanshinone IIA has been proven to enhance the activities of superoxide dismutase and catalase by activating the nuclear factor erythroid 2-related factor 2 (Nrf2)/antioxidant response element (ARE) pathway, effectively scavenging ROS and protecting vascular endothelial cells from oxidative damage (16). Combined with the significant regression of xanthine and hypoxanthine observed in this study, it is speculated that Tanshinone IIA may protect microvascular endothelial integrity and delay the ischemic necrosis process by improving the local hypoxic state of the femoral head and inhibiting the oxidative stress generated by purine catabolism. At the level of energy metabolism, the abnormal elevation of 2-oxopentanedioic acid and succinic semialdehyde may reflect disturbances in TCA cycle flux and GABA shunt metabolism, respectively. Since succinic semialdehyde is converted to succinate and enters the TCA cycle, these changes may converge on mitochondrial energy metabolism (29). The extensive enrichment of the 2-oxocarboxylic acid metabolism and alanine/aspartate/glutamate metabolism pathways may reflect hormone-induced mitochondrial dysfunction (30). Recent research using ¹³C metabolic flux analysis has confirmed that Tanshinone IIA can restore the activity of mitochondrial respiratory chain complexes, reverse aberrant glutamine anaplerosis, and enhance cellular tolerance to hypoxia (31). Furthermore, the recovery of pantothenate (a precursor of coenzyme A) levels and the enrichment of the pantothenate and CoA biosynthesis pathway suggest that Tanshinone IIA may improve fatty acid β-oxidation efficiency and reduce abnormal lipid accumulation in the marrow cavity, which correlates with the morphological changes of reduced fatty infiltration observed by HE staining (32).

It is noteworthy that pyruvate and glutamic acid were decreased in the Tanshinone IIA group compared with the model group. Pyruvate is a critical regulatory metabolite at the crossroads of cytoplasmic glycolysis and mitochondrial oxidative phosphorylation, and its utilization by mitochondria is tightly controlled by the mitochondrial pyruvate carrier and pyruvate dehydrogenase, which converts pyruvate to acetyl-CoA to fuel the TCA cycle (33). Therefore, a further decline in pyruvate may indicate enhanced entry into the TCA cycle and increased oxidative phosphorylation. Similarly, glutamate dehydrogenase catalyzes the oxidative conversion of glutamate to α-ketoglutarate, providing the TCA cycle with an anaplerotic intermediate and supporting energy production via oxidative phosphorylation (34). Tanshinone IIA has been reported to stimulate mitochondrial NADH oxidation and restore respiratory chain activity in mitochondria, and to modulate mitochondrial energy metabolism by regulating glutamine metabolic reprogramming (31, 35). Thus, the further decreases in pyruvate and glutamic acid are consistent with a metabolic remodeling effect in which these substrates are more actively consumed to support mitochondrial energy metabolism, rather than a simple return to baseline.

Proteomics showed that DEPs between the model and control groups were significantly enriched in signaling pathways including nitrogen metabolism, Hippo, TGF-β, Ras, and Wnt. Among these, the Hippo and Wnt pathways are particularly critical in SONFH—they jointly regulate the balance between osteogenic and adipogenic differentiation of BMSCs and bone homeostasis (36). After Tanshinone IIA intervention, aminoacyl-tRNA biosynthesis and lipid metabolism-related pathways became the main enriched items, indicating that its effects may involve the modulation of protein synthesis efficiency and lipid metabolism reprogramming (37). Pearson correlation analysis identified Bmp6, Smoc2, Ihh, and Enpp2 as core nodes in the protein-metabolite association network. These proteins have well-defined functions in bone development and angiogenesis: BMP6 promotes osteogenic differentiation and induces vascular endothelial growth factor (VEGF) expression through the Smad pathway (38); SMOC2, an extracellular matrix protein, can enhance BMP2 signaling and promote vascularized bone healing (39); Ihh is a key ligand of the Hedgehog signaling pathway, coordinating endochondral ossification and vascular invasion (40); ENPP2 participates in angiogenesis and osteoblast differentiation by producing lysophosphatidic acid (41). It is noteworthy that a recent study revealed a novel mechanism of Tanshinone IIA in SONFH—alleviating osteocyte pyroptosis by inhibiting the endoplasmic reticulum stress-mediated IRE1α/TXNIP/NLRP3 pathway, thereby restoring bone mass (42). Osteocyte pyroptosis is an important form of cell death in SONFH; activation of the NLRP3 inflammasome releases IL-1β and IL-18, further aggravating local inflammation and vascular injury (43). The TGF-β and Wnt pathways enriched in our proteomic data interact with this mechanism, and Tanshinone IIA may block the cascade of “glucocorticoid → oxidative stress → endoplasmic reticulum stress → pyroptosis” through multi-level intervention. The significant correlations between these proteins and purine metabolites and organic acids further suggest that Tanshinone IIA may simultaneously regulate bone development signaling, angiogenesis, and metabolic enzyme activity to achieve synergistic improvement of bone tissue repair and systemic metabolic homeostasis. This multi-node regulatory pattern provides comprehensive advantages over single-target drugs.

This study has several limitations. The sample size was relatively small (n = 6 per group for metabolomics; n = 5 per group for proteomics; n = 3 per group for Micro-CT and histology), which may limit statistical power and the generalizability of the findings. First, fecal metabolomics reflects systemic host–microbiota metabolism but cannot directly represent local ischemia/hypoxia in the femoral head. Although serum, bone tissue, synovial fluid, and bone marrow are more directly related to the local microenvironment, their collection is invasive, limited in volume, and unsuitable for repeated sampling in small animals; future studies should validate local metabolic changes using these samples. Second, no positive drug control was included; therefore, the relative efficacy of Tanshinone IIA compared with existing hip-preserving drugs could not be determined. Future studies should include positive controls, such as low molecular weight heparin or bisphosphonates. Third, network centrality analysis was not performed; candidate proteins were identified based on correlation analysis and literature evidence and should be regarded as hypothesis-generating rather than definitive hub proteins. Moreover, the causal contributions of candidate proteins such as Bmp6 and Enpp2 require confirmation through tissue-specific gene knockout or pharmacological blocking experiments. Fourth, trabecular number (Tb.N) was not assessed in the present Micro-CT analysis, and HE-staining quantification was limited to trabecular area; adipocyte-related metrics and empty lacunae rate were not measured due to archival section constraints. These bone-related indices will be incorporated in future investigations.

Notably, although Tanshinone IIA exhibits prominent pharmacological activities, its intrinsic poor aqueous-solubility constrains its direct local application at necrotic bone sites. Local delivery via injectable biodegradable hydrogel has been reported to achieve high local drug concentrations at osteonecrotic lesions, representing an attractive localized clinical translation strategy (44). In the present study, Tanshinone IIA was administered as an aqueous suspension by oral gavage because of its poor aqueous solubility. This formulation may have limited oral bioavailability and systemic exposure, which should be considered when interpreting the observed effects. Future studies should optimize delivery systems, such as nanoformulations or injectable biodegradable hydrogels, to improve local and systemic drug exposure.

In summary, this study provides a mechanistic map of Tanshinone IIA against SONFH from a multi-omics integration perspective: it not only corrects abnormal BMSC differentiation, inhibits osteocyte pyroptosis and osteoclast activation by regulating multiple signaling pathways such as Wnt/β-catenin, PPARγ, TGF-β, and IRE1α/TXNIP/NLRP3, but also achieves dual improvements in bone protection and metabolic homeostasis by ameliorating purine metabolism disorders caused by ischemia-hypoxia, restoring TCA cycle flux, and modulating key protein nodes. These findings provide solid experimental evidence for the development of Tanshinone IIA as a candidate drug for SONFH.

## 5 Conclusion

This study systematically evaluated the interventional effects of Tanshinone IIA on a rat model of SONFH using metabolomics combined with proteomics. The results demonstrate that Tanshinone IIA can significantly ameliorate femoral head bone microarchitecture destruction, alleviate fatty infiltration, and inhibit osteoclast activation. Metabolomics suggested that Tanshinone IIA partially reverses the abnormal accumulation of purine metabolites and metabolic intermediates related to the TCA cycle and GABA shunt, including 2-oxopentanedioic acid and succinic semialdehyde, and may induce metabolic remodeling rather than simple normalization. Proteomics suggested associations with signaling pathways such as Wnt, TGF-β, and Hippo, and candidate proteins including Bmp6 and Ihh were correlated with metabolite changes. In conclusion, Tanshinone IIA may be associated with potential multi-target effects at three levels—bone protection, metabolic alterations, and signaling network associations—providing preliminary multi-omics evidence for further investigation in SONFH.

## Data Availability

The contributions originally presented in this study are included in the article and **Supplementary Material**. Further inquiries can be directed to the corresponding authors.
